# Integrated child development service (ICDS) coverage among severe acute malnourished (SAM) children in India: A multilevel analysis based on national family health survey-5

**DOI:** 10.1371/journal.pone.0294706

**Published:** 2024-02-08

**Authors:** Ritankar Chakraborty, William Joe, Udaya ShankarMishra, Sunil Rajpal

**Affiliations:** 1 Department of Bio-Statistics and Epidemiology, International Institute for Population Sciences, Mumbai, Maharashtra, India; 2 Population Research Centre, Institute of Economic Growth, Delhi University Enclave (North Campus), Delhi, India; 3 Department of Economics, FLAME University, Pune, Maharashtra, India; Indian Institute of Dalit Studies (IIDS), INDIA

## Abstract

Severe acute malnutrition (SAM) can be fatal for children, and potentially limit their cognitive and physical growth. The last three National Family Health Survey (NFHS) in India shows an increase in the prevalence of SAM among under-five children. Given the specific mandates under ICDS (Integrated Child Development Service) for SAM children, it is important to validate the coverage efficiency of ICDS on SAM children. This paper examines a possible association between the coverage efficiency of ICDS on SAM children. The study further aims to identify the determinants of ICDS service utilization among SAM children. We used data from the fifth round of the National Family Health Survey. Descriptive statistics was used to estimate the SAM coverage under ICDS. Multilevel Logistic Regression was used to identify the determinants of ICDS service utilization among SAM children. The burden of SAM is higher among older children (3+ age). Coverage of ICDS was more among younger children and the poorest households in the rural areas. Results from multilevel logistic regression showed that age had a significant relationship with the outcome variable. SAM children living in the rural areas had a significantly higher odds of being covered under ICDS service (OR 1.57; CI: (1.35, 1.82)) than their urban counterparts. Pregnant and lactating mothers who received ICDS services were significant determinants of SAM coverage under ICDS. There is no evidence that ICDS is more efficient in identifying and covering SAM children than non-SAM children. Despite special provisioning in place for SAM children, coverage of different ICDS services was similar to that of non-SAM children, and were in fact lower than non-SAM children for some categories. The study suggests that improving coverage of ICDS services among pregnant and lactating mothers would increase the coverage of ICDS services among SAM children.

## Introduction

Severe Acute Malnutrition (SAM) among children aged less than 5 years is defined in terms of any of the following three diagnostic criteria: a) weight-for-height Z-score < -3 SD of WHO (World Health Organization) child growth standards, known as severe wasting [1]; b) presence of bilateral pitting oedema [2]; or c) a MUAC (mid-upper-arm circumference) < 115 mm [3]. Severe acute malnutrition can be fatal for children, and those who survive are maimed both intellectually and physically. More than 5,00,000 deaths of children under 5 years of age are attributable to severe wasting or a WHZ (Weight-for-height z) score < -3 SD annually [4]. Globally, 20 million children suffer from SAM, leading to the deaths of 1 million each year making it one of the leading causes of death among under-five children [5]. A child suffering from SAM has 10 times greater likelihood of dying as against their well-nourished counterparts. The case fatality rate for SAM among children under 5 years of age varied from 20% to 50% across some centers till the end of the 20^th^ century [6]. Sub-Saharan Africa, Asia and Latin America have the highest burden of SAM children in the world.

India is burdened with more than 8 million SAM children [7–9]. The country accounts for nearly 0.6 million deaths and 24.6 million DALYs (Disability adjusted life years), all attributable to stunting and severe wasting [7, 10]. Previous rounds of National Family Health Survey (NFHS) in India witnessed an increase in the prevalence of SAM among under-five children. The prevalence of SAM was at 6.4% in 2005–06 [11], 7.5% in 2015–16 [12] and 7.7% in 2019–21 [13]. The Millennium Development Goals (2000) and Sustainable Development Goals (2015) aims at reducing hunger and undernourishment among children. SDG 2 of “Zero Hunger” aimed to end all forms of hunger and malnutrition by 2030-especially among children under 5 years of age by ensuring sufficient and nutritious food. However, India is yet to progress well to achieve the SDG target by 2030. According to NITI Aayog’s SDG Index 2020–21, India had a score of 47 out of 100 in 2020 on Goal 2 indicating that the performance of the country leaves much to be desired [14].

Since the 1970s, India has been combating the problem of child malnutrition. The flagship Integrated Child Development Service (ICDS) scheme was launched in 1975. The recent National Nutrition Mission (NNM) launched in 2017 have shown ever increasing intent to reduce child malnourishment in the country. The NNM widely referred to as the POSHAN Abhiyaan aims at improving programmatic performance and inter-departmental coordination to escalate the efforts to reduce child undernutrition [15–20]. The ICDS scheme focuses on children under 6 years of age, pregnant and lactating mothers and women in the age group of 15–44 years with services including supplementary nutrition, immunization, health check-ups, referrals, nutrition and health education and pre-school education [21]. These services are provided at “*Anganwadi*” centres (courtyard shelters) through community health workers (Anganwadi workers). Under ICDS, the SAM children aged 6–72 months are entitled to receive 800 Kcal and 20–25 grams of protein per day as supplementary nutrition.

Evaluation of this programme as regard to its implication on nutritional make up of children in general has been a continuous endeavour that justifies its continuation and diversification over time. However, the comprehensive scheme of ICDS has been performing with varying efficiency across regions to deliver on its targeted outcomes. Given its wider provisioning domain, there remains every scope to assess its impact on adverse outcome like severe and acute malnutrition that is yet to be exposed in literature.

Given the specific mandates under ICDS for SAM children, it is important that the scheme cover all these children. The ICDS is expected to address the specific need of SAM children with interventions by Anganwadi workers (AWWs) in terms of dietary provisioning and counselling support. Therefore, it becomes pertinent to examine the association between ICDS coverage and the share of SAM beneficiaries. More than a systematic reduction in the prevalence of child undernourishment, a faster reduction in SAM children should perhaps be the focus of ICDS. Nutritional makeup of children and its association with the levels of ICDS coverage may well be in expected direction, but unless there is a focus on SAM children, there is every likelihood of a failure in arresting nutrition-linked mortality.

Therefore, this study examines coverage efficiency of ICDS on SAM children. Existing studies on ICDS coverage have mainly focused on service utilization by pregnant and lactating mothers, and by children aged 0–72 months [22, 23]. But none of these studies have any explicit attention on the coverage status of SAM children. At the same time, there is no systematic analysis on the state-wise performance in coverage of ICDS among the SAM children as well as the equity in coverage by socio-economic background. Since the ICDS has a specific focus on SAM, it is important to validate the coverage efficiency of ICDS on SAM children. The second objective of this study is to find out the determinants of ICDS service utilization among SAM children. Some studies have tried to assess the impact of ICDS on child undernutrition using previous rounds of NFHS [24–29], but none have explored the factors that determine utilization of ICDS services among SAM children.

## Data and methods

### Survey data and study population

**Fig 1:**
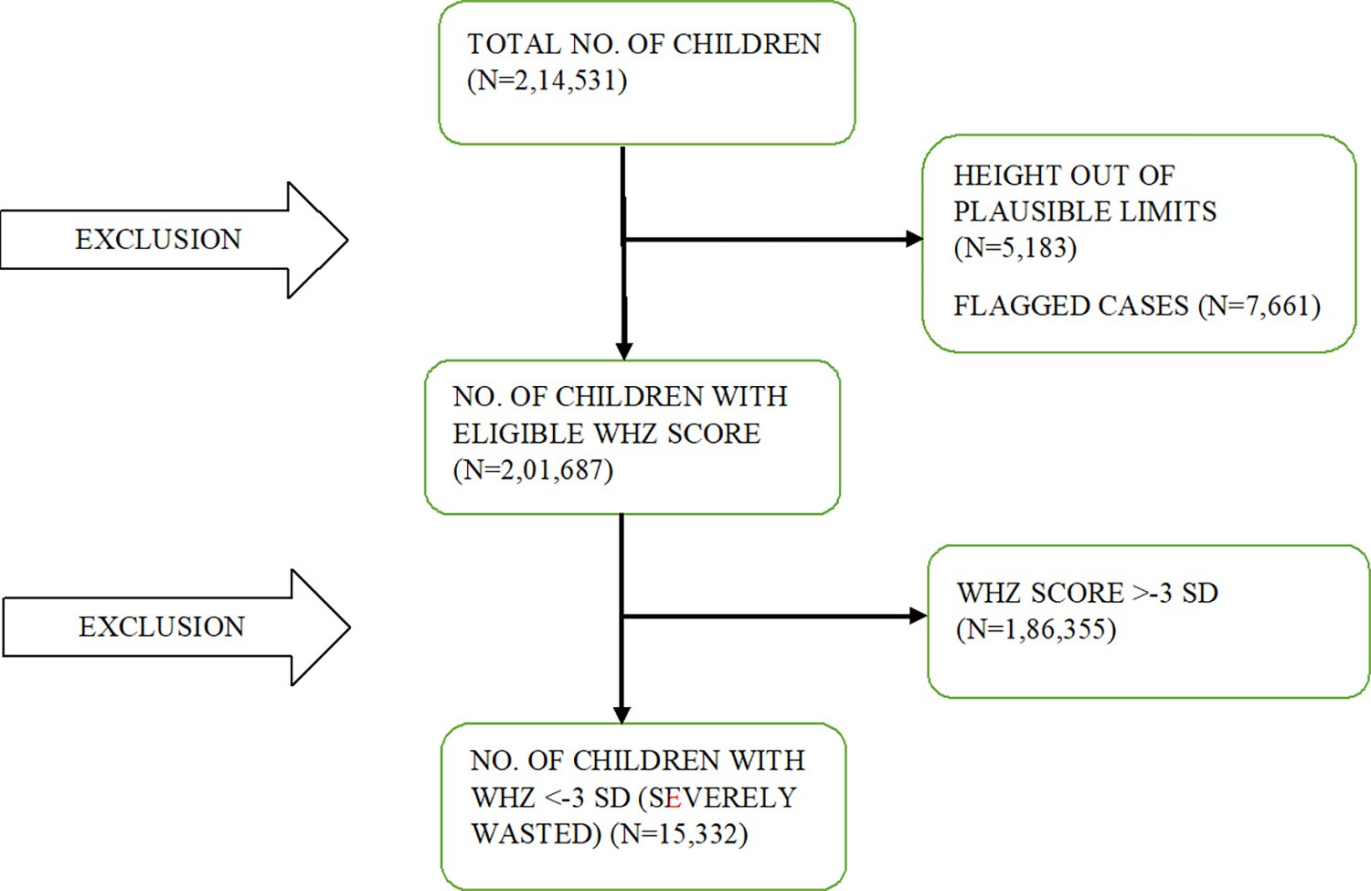
Flowchart for sample selection.

The study uses data from the 5^th^ round of the National Family Health Survey (NFHS-5) conducted in 2019–21. The sample for NFHS-5 was designed to provide estimates of all key maternal and child health and nutrition indicators at the national and state levels, as well as estimates for selected indicators at the district level. The sample size of 6,10,000 households across India was selected in order to produce reliable indicator estimates. A two-stage sampling procedure was adopted for both the rural and urban areas. In the rural areas, villages were selected as the Primary Sampling Units (PSUs) at the first stage (selected with probability proportional to size), followed by a random selection of 22 households in each PSU at the second stage. In the urban areas, Census Enumeration Blocks (CEBs) were selected at the first stage and a random selection of 22 households in each CEB at the second stage. A complete mapping and household listing operation was performed on the first-stage units from which the households were selected in the second stage in both the urban and rural areas. This study included only those children under 5 years with Weight-for-Height (WHZ) score < -3 SD of WHO child growth standards. NFHS 5 collected information on 2,14,531 children. Out of those, there were 7,661 flagged cases for WHZ and 5,183 cases out of plausible limits. After excluding all the invalid non-flagged weight-for-height z scores, a final analytic sample of 15, 332 children with WHZ score < -3 SD were used for the primary analysis. Fig 1 gives the flowchart for sample selection.

### Outcome variable

The primary outcome variable for the analysis was: whether the child received benefits (any) from anganwadi/ICDS centre in the last 12 months? (Survey question: During the last 12 months, has (name of child) received any benefits from the anganwadi?). Receiving any benefits from the Anganwadi includes receiving either food, immunization, health check-ups, pre-school education or having weight measured. The outcome variable was recoded as a binary variable (yes = 1/no = 0) for the primary analysis.

### Correlates

Age of the child was recoded into five categories (<1 year, 1–2 years, 2–3 years, 3–4 years and 4–5 years). Birth Order was recoded as- Birth Order 1–2, Birth Order 3–4 and Birth Order 4+. Religion was categorized into four groups-Hindus, Muslims, Christians and Others. Households were categorized based on the social groups- Scheduled Castes (SCs), Scheduled Tribes (STs), Other Backward Classes (OBCs) and Others. Preceding birth interval was categorized as- <24 months, 24–35 months, 36–47 months, 48–59 months and 60+ months. Place of delivery was divided into 3 categories: Non-institutional, Public institutional and Private institutional. Wealth index was taken as the proxy indicator for the household’s income. In NFHS, Wealth Index was created by Principal Component Analysis (PCA) on household wealth and assets and was divided into five quintiles- poorest, poorer, middle, richer and richest [12]. Whether the mother received any Anganwadi benefits during pregnancy and during breastfeeding were taken as binary variables (yes/no). Exposure to mass media was created as a binary variable (yes/no) by combining three variables- use of radio, television and newspaper. Place of residence (rural/urban), sex of the child (male/female), maternal education (no education/ primary/ secondary/ higher) were the other categorical predictors used in the analysis. The correlates were considered based on existing literature [22, 30].

### Statistical analysis

The study uses descriptive statistics to inform on the prevalence of severe wasting among urban and rural children based on the socio-economic and demographic background characteristics. Cross tabulation was performed to estimate the percentage of SAM children who received any ICDS benefits across various states of India. Coverage of different ICDS services among SAM and non-SAM children and among mothers of SAM and non-SAM children based on background characteristics were also computed. Concentration Index (CI) was calculated for estimating the socio-economic inequality in coverage of ICDS services among SAM children across the states. The results of the descriptive analysis are presented in graphical and tabular forms.

To understand how service utilization among SAM children is associated with household’s socio-economic and demographic correlates, we fit a multilevel logistic regression with children considered as level 1 and the random effects introduced for PSU (level 2), district (level 3) and state (level 4). The random effects of the PSU, district and state were estimated using the *melogit* command in Stata (version 16.0) [31]. Two models were fitted for the outcome variable. The first model, called the empty/null model comprised of no exposure variables. It focused on decomposing the total variance into the PSU, district and state, which is a useful measure to determine the extent of cluster variation in utilization of ICDS services. The Variance Partitioning Coefficient (VPC) was calculated to measure the extent of variation in utilization of ICDS services that can be attributed to PSU, district and state levels [32]. While the variance at the state level would call for greater state level policy focus, variations attributed to lower level would entail strengthening of program monitoring and implementation. The second model was adjusted for the socio-economic and demographic correlates. Odds ratios and 95% CIs were reported in the table for the second model.

The regression equation of the four-level model (second model) is as follows:

$$logit(\pi_{ijkl})=\log(\frac{\pi_{ijkl}}{1-\pi_{ijkl}})=\beta_{0jkl}+\beta_{1}x_{1ijkl}+\beta_{2}x_{2ijkl}+\cdots +\beta_{n}x_{nijkl}+{(f}_{0l}+v_{0kl}+u_{ojkl})$$

Here, $\pi_{ijkl}=p(y_{ijkl}=1)$ is the probability that a severely wasted child (i) in PSU (j), from district (k) in state (l) has received ICDS service (any) in the last 12 months. Here *y*_*ijkl*_ is equal to 1 if the child received ICDS benefits and is equal to 0 if child did not. *β*_0*jkl*_ is the random intercept term at PSU, district and state levels.

The advantage of this method is that one error term is generated at each level, which allows us to isolate the residual variances at each level. The random effects inside the bracket were the residual terms for the state *l* (*f*_*0l*_), district *k* (*v*_*0kl*_), and PSU *j* (*u*_*0jkl*_). All the three residuals were assumed to be independent and follow the standard normal distribution with mean 0 and variance *σ*^2^*f*_0_, *σ*^2^*v*_0_, and *σ*^2^*u*_0_.

The Variance Partitioning Coefficient (VPC) at each level was obtained by dividing the variation at that level divided by the total variation.

So, the VPC at the state level was calculated as: ${VPC}_{state}=\frac{\sigma^{2}f_{0}}{\sigma^{2}f_{0}+\sigma^{2}v_{0}+\sigma^{2}u_{0}}$

Similarly, the VPC at the district level was calculated as: ${VPC}_{district}=\frac{\sigma^{2}v_{0}}{\sigma^{2}f_{0}+\sigma^{2}v_{0}+\sigma^{2}u_{0}}$, and

the VPC at the PSU level was calculated as: ${VPC}_{psu}=\frac{\sigma^{2}u_{0}}{\sigma^{2}f_{0}+\sigma^{2}v_{0}+\sigma^{2}u_{0}}$

Also, we fitted another multilevel logistic regression to identify the determinants of ICDS service utilization among all children. Whether the child received any ICDS service was taken as the outcome variable. This was done in order to compare the effects of the correlates between ICDS coverage for all children and ICDS coverage for SAM children.

## Results

**Fig 2:**
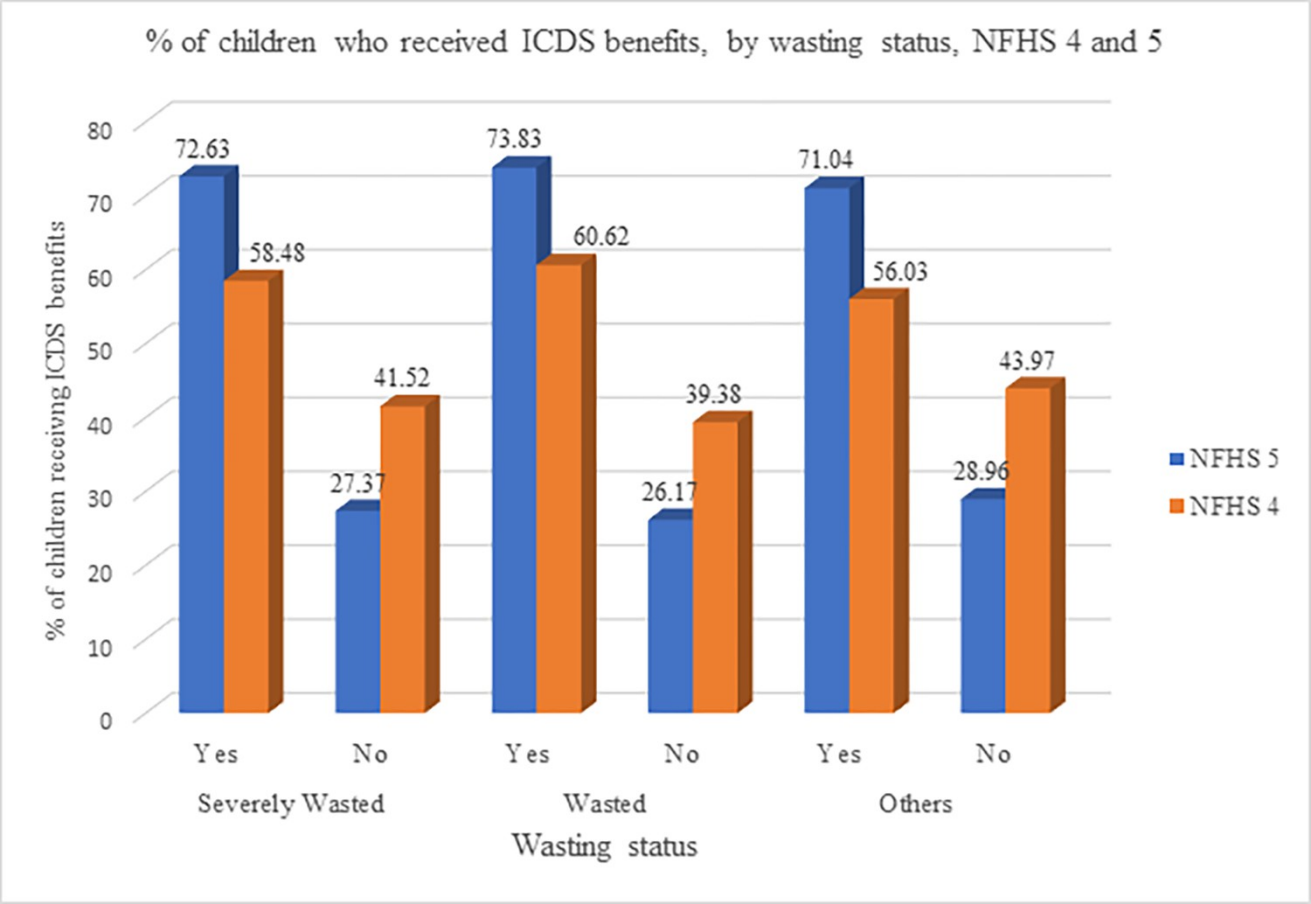
Percentage of children who received ICDS benefits, by wasting status, India NFHS 4 and 5.

Between NFHS 4 (2015–16) and NFHS 5 (2019–21), there is an increase in the percentage of children who received any services and benefits under ICDS scheme (Fig 2). Among the severely wasted children, 73% children received any kind of ICDS benefit in NFHS 5 as compared to 58% in NFHS 4. The level of coverage was 74% among children with any wasting vis-à-vis 71% among those above the wasting threshold.

Table 1 shows ICDS service utilization among SAM children by place of residence and background characteristics. Among the SAM children, those below 3 years of age had received higher coverage under ICDS (70%) than those above 3 years of age (30%). SAM coverage under ICDS was similar in the rural and urban areas for children both below and above 3 years of age. Male children have a slightly higher coverage of ICDS services (54%) than females (46%) with no such difference between the urban and rural areas. Approximately, 55% of the SAM children whose mothers had secondary education received ICDS benefits in the urban areas as compared to only 50% in the rural areas. ICDS service utilization among SAM children was highest among the Other Backward Classes (OBCs) both in the rural and urban areas. In the rural areas, 23% of those receiving any ICDS benefits belonged to the Scheduled Caste (SC), whereas 25% and 40% belonged to Scheduled Tribe (ST) and Other Backward Classes (OBC) respectively. Majority of the SAM children who received any ICDS benefits in the rural areas belonged to the poorest wealth quintile (37%), followed by those belonging to the poorer (27%), middle (19%), richer (12%) and richest (5%) wealth quintiles. But a complete opposite of this gradient was observed in the urban areas, where SAM coverage under ICDS was best among the richest (32%) and richer households (32%) as compared to the poorer (11%) and poorest households (4%).

**Table 1:** ICDS service utilization among SAM children by place of residence and background characteristics, NFHS 5.

	Place of residence→	Urban (n, %)	Rural (n, %)	Urban (n, %)	Rural (n, %)	All (n, %)
Background Characteristics↓	Received ICDS benefit→	No		Yes		
Age of child						
<3 years		713 (62.38)	1862 (60.99)	1276 (70.23)	6579 (70.60)	10430 (68.03)
3–5 years		430 (37.62)	1191 (39.01)	541 (29.77)	2740 (29.40)	4902 (31.97)
Total		1143 (100)	3053 (100)	1817 (100)	9319 (100)	15332 (100)
Sex of the child						
Male		601 (52.58)	1680 (55.03)	976 (53.71)	5064 (54.34)	8321 (54.27)
Female		542 (47.42)	1373 (44.97)	841 (46.29)	4255 (45.66)	7011 (45.73)
Total		1143 (100)	3053 (100)	1817 (100)	9319 (100)	15332 (100)
Maternal Education						
No Education		123 (10.76)	981 (32.13)	227 (12.49)	2521 (27.05)	3852 (25.12)
Primary		100 (8.75)	421 (13.79)	177 (9.74)	1333 (14.30)	2031 (13.25)
Secondary		556 (48.64)	1372 (44.94)	1000 (55.04)	4700 (50.43)	7628 (49.75)
Higher		364 (31.85)	279 (9.14)	413 (22.73)	765 (8.21)	1821 (11.88)
Total		1143 (100)	3053 (100)	1817 (100)	9319 (100)	15332 (100)
Social Group						
Scheduled Caste		181 (17.19)	537 (19.17)	368 (21.45)	2043 (23.15)	3129 (21.74)
Scheduled Tribe		113 (10.73)	743 (26.53)	154 (8.97)	2247 (25.46)	3257 (22.63)
Other Backward Class		433 (41.12)	1104 (39.41)	813 (47.38)	3510 (39.78)	5860 (40.71)
Others		326 (30.96)	417 (14.89)	381 (22.20)	1024 (11.60)	2148 (14.92)
Total		1053 (100)	2801 (100)	1716 (100)	8824 (100)	14394 (100)
Wealth Quintile						
Poorest		63 (5.51)	1187 (38.88)	82 (4.51)	3424 (36.74)	4756 (31.02)
Poorer		101 (8.84)	833 (27.28)	204 (11.23)	2522 (27.06)	3660 (23.87)
Middle		184 (16.10)	494 (16.18)	379 (20.86)	1823 (19.56)	2880 (18.78)
Richer		309 (27.03)	371 (12.15)	575 (31.65)	1112 (11.93)	2367 (15.44)
Richest		486 (42,52)	168 (5.50)	577 (31.76)	438 (4.70)	1669 (10.89)
Total		1143 (100)	3053 (100)	1817 (100)	9319 (100)	15332 (100)

Table 2 gives the SAM prevalence along with the SAM coverage under ICDS and the Concentration Index (CI) of SAM coverage under ICDS for NFHS 4 and NFHS 5. While some states and UTs like Assam, Bihar, Lakshadweep, Maharashtra, Nagaland, Tripura, Uttar Pradesh and Telangana have shown sharp increase in the burden of SAM children, other states like Arunachal Pradesh, Chhattisgarh, Jharkhand, Karnataka, Madhya Pradesh, Meghalaya, Puducherry, Tamil Nadu and Uttarakhand have shown significant declines in SAM prevalence. SAM coverage under ICDS has increased in almost all states and UTs, except in Chandigarh and Punjab. The highest increase in SAM coverage under ICDS was observed in Uttar Pradesh, Delhi, Karnataka, Jammu and Kashmir, Rajasthan, Haryana and Goa. The North-Eastern states of Sikkim, Nagaland, Mizoram and Manipur have also performed better in improving SAM coverage under ICDS. A reduction in wealth-based inequalities have also been observed in most of the states and UTs.

**Table 2:** SAM prevalence and concentration index value for ICDS coverage of SAM children, by state, NFHS 4 & 5.

States	SAM %		Any ICDS benefit %		Con Index of SAM coverage under ICDS (SE)	
	NFHS 4	NFHS 5	NFHS 4	NFHS 5	NFHS 4	NFHS 5
**STATES ↓**						
Andhra Pradesh	4.56	5.99	80.31	81.41	-0.087 (0.025)	-0.029 (0.026)
Arunachal Pradesh	7.65	6.52	23.3	37.83	-0.172 (0.065)	0.004 (0.054)
Assam	6.15	9.02	61.85	66.92	-0.002 (0.025)	0.025 (0.019)
Bihar	6.94	8.8	52.19	57.49	-0.015 (0.017)	-0.028 (0.015)
Chhattisgarh	8.37	7.57	86.85	84.9	-0.015 (0.015)	-0.019 (0.015)
Goa	9.43	7.52	53.22	73.61	0.001 (0.084)	0.001 (0.084)
Gujarat	9.67	10.54	64.62	71.15	-0.059 (0.023)	-0.055 (0.084)
Haryana	9.01	4.34	49.39	71.68	0.005 (0.029)	0.025 (0.027)
Himachal Pradesh	4.04	4.04	65.82	84.93	-0.045 (0.048)	0.024 (0.025)
Jharkhand	11.26	9.09	64.77	74.39	-0.052 (0.016)	-0.044 (0.015)
Karnataka	10.33	8.42	60.29	87.13	-0.107 (0.028)	-0.008 (0.012)
Kerala	6.49	5.76	56.07	63.27	-0.120 (0.044)	0.043 (0.044)
Madhya Pradesh	9.18	6.43	71.48	83.53	-0.004 (0.01)	-0.017 (0.012)
Maharashtra	9.45	10.87	47.16	57.74	-0.141 (0.03)	-0.160 (0.038)
Manipur	2.21	3.41	19.18	40.32	-0.076 (0.114)	-0.026 (0.082)
Meghalaya	6.36	4.71	47.91	55.46	-0.050 (0.042)	0.064 (0.036)
Mizoram	2.18	4.88	53.29	66.63	0.050 (0.08)	0.017 (0.05)
Nagaland	4.23	7.84	33.67	55.55	0.025 (0.077)	-0.123 (0.048)
Odisha	6.4	6.04	83.51	92.28	-0.001 (0.014)	-0.010 (0.009)
Punjab	5.58	5.58	68.32	57.27	-0.116 (0.029)	-0.119 (0.043)
Rajasthan	8.56	7.58	42.5	65.67	-0.044 (0.022)	-0.042 (0.017)
Sikkim	6	6.65	53.61	77.07	-0.185 (0.077)	-0.114 (0.086)
Tamil Nadu	7.8	5.47	61.08	80.99	-0.118 (0.022)	-0.039 (0.019)
Telangana	4.54	8.54	66.57	79.02	-0.182 (0.044)	-0.049 (0.022)
Tripura	6.2	7.38	62.91	69.88	-0.084 (0.051)	-0.044 (0.038)
Uttar Pradesh	6.03	7.23	42.62	74.41	-0.065 (0.018)	-0.019 (0.009)
Uttarakhand	8.91	4.72	68.33	86.28	-0.046 (0.025)	-0.004 (0.028)
West Bengal	6.4	7.2	78.1	81.92	-0.043 (0.021)	-0.036 (0.015)
**UTs ↓**						
Andaman	7.13	4.82	52.76	61.29	-0.362 (0.066)	0.013 (0.133)
Chandigarh	3.47	2.33	52.28	45.61	-0.370 (0.073)	0.033 (0.419)
Dadra & Nagar Haveli	10.99	4.3	46.99	71.07	-0.083 (0.095)	0.031 (0.061)
Daman & Diu	12.08	NA	40.42	NA	-0.060 (0.148)	NA
Delhi	4.67	4.82	22.26	53.4	-0.086 (0.164)	-0.051 (0.065)
Jammu & Kashmir	5.57	5.57	23.13	50.05	-0.093 (0.072)	-0.060 (0.034)
Ladakh	NA	9.32	NA	51.82	NA	0.129 (0.075)
Lakshadweep	2.99	8.71	55.75	64.09	0.307 (0.136)	-0.065 (0.092)
Puducherry	7.82	3.73	58.54	79.77	-0.194 (0.056)	-0.142 (0.033)

As of NFHS 5, among the states, Maharashtra had the highest prevalence of SAM children (10.8%) followed by Gujarat (10.5%) and Jharkhand (9.0%). The North-Eastern states of Manipur, Meghalaya, Mizoram and the Northern states of Haryana and Himachal Pradesh are the least burdened with SAM children. There are 10 states and UTs combined that have a prevalence of SAM children greater than the national average of 7.7%. SAM coverage under ICDS is highest in Odisha (92.28%), followed by Karnataka (87.13%), Uttarakhand (86.28%), Himachal Pradesh (84.93%) and Chhattisgarh (84.90%). Arunachal Pradesh is at the bottom with only 38% of the SAM children being covered under ICDS. The negative Concentration Index values show that in most of the states and UTs, SAM coverage under ICDS is pro-poor in nature. States like Maharashtra (-0.16), Nagaland (-0.123), Punjab (-0.119) and Sikkim (-0.114) have the highest values of Concentration Index, though the values being close to zero indicate that the extent of wealth-based inequality in SAM coverage under ICDS is low.

**Fig 3:**
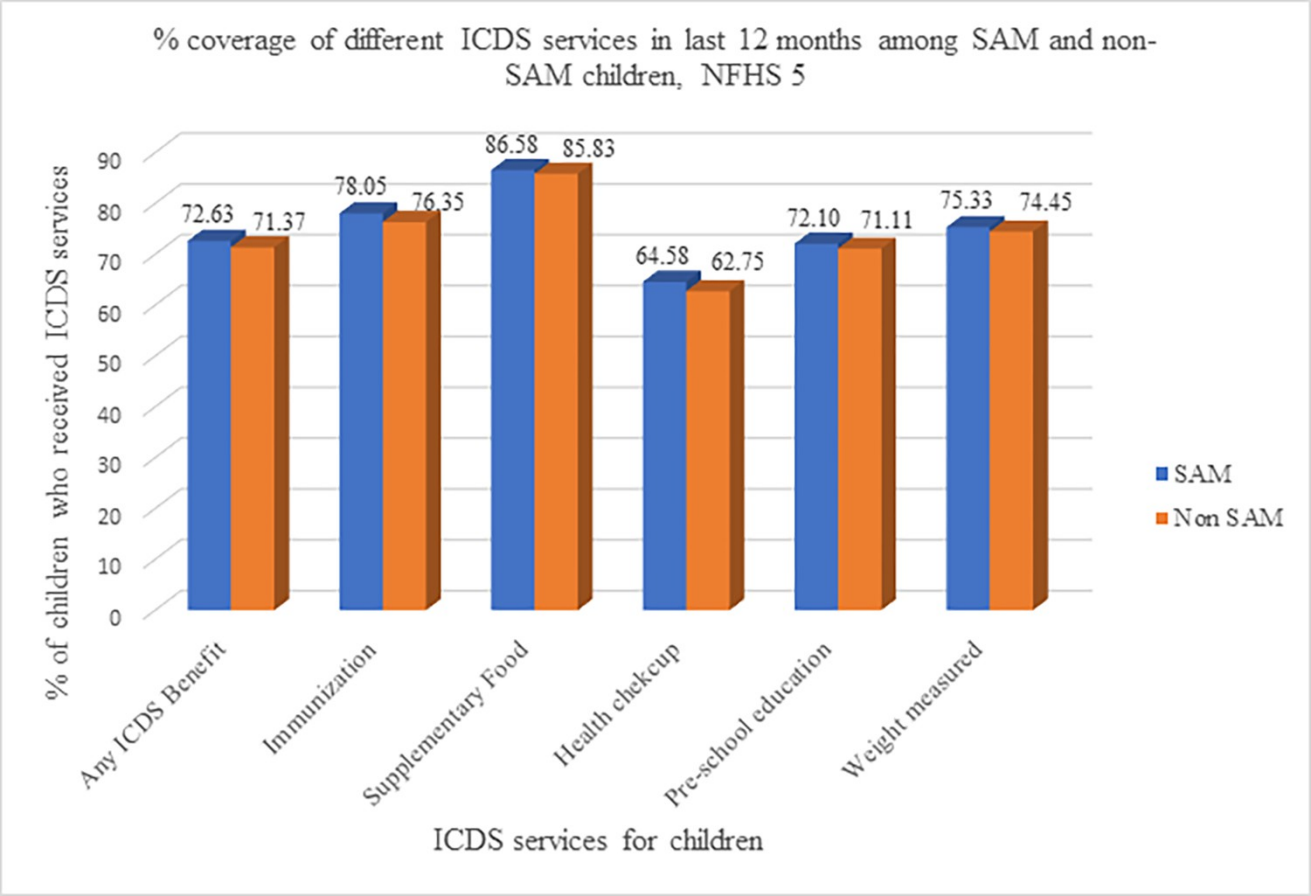
Percentage coverage of different ICDS services among SAM and Non-SAM children, NFHS 5.

Fig 3 shows the percentage coverage of different ICDS services in last 12 months among SAM and non-SAM children. The coverage of the different ICDS services were almost identical among both SAM and non-SAM children.

Table 3 gives the coverage of different ICDS services, namely Immunization, Food supplements, health check-up, pre-school education and measurement of weight among SAM and non-SAM children by background characteristics. The coverage of the different ICDS services were almost similar for both SAM and non-SAM groups, though the coverage among the SAM children were lower than non-SAM children for those aged 3–5 years, female children, mothers with secondary and higher education, belonging to Other Backward Classes, belonging to middle, richer or richest households and those living in the urban areas. The results from Table 3 suggests that in spite of special provisioning for SAM children, coverage of different ICDS services was similar to that of non-SAM children, and worse off for some categories.

**Table 3:** Coverage of different ICDS services among SAM and Non-SAM children by background characteristics.

Type of ICDS service →	Received Immunization		Received Food		Had health check-up		Received pre-school education		Had weight measured	
Type of child →	SAM (n,%)	Non- SAM (n,%)	SAM (n,%)	Non- SAM (n,%)	SAM (n,%)	Non- SAM (n,%)	SAM (n,%)	Non- SAM (n,%)	SAM (n,%)	Non- SAM (n,%)
Background Characteristics ↓										
Age of child										
< 3 years	6,321 (72.72)	64,831 (63.84)	6,766 (70.18)	69,918 (61.24)	5,063 (70.40)	51,489 (61.70)	5,289 (65.87)	53,194 (56.24)	5,865 (69.91)	60,438 (61.03)
3–5 years	2,371	36,718	2,875	44,244	2,129	31,968	2,740	41,389	2,524	38,592
	(27.28)	(36.16)	(29.82)	(38.76)	(29.60)	(38.30)	(34.13)	(43.76)	(30.09)	(38.97)
Total	8,692	101,549	9,641	114,162	7,192	83,457	8,029	94,583	8,389	99,030
	(100)	(100)	(100)	(100)	(100)	(100)	(100)	(100)	(100)	(100)
Sex of child										
Male	4,694	51,957	5,203	58,248	3,876	42,499	4,343	48,037	4,522	50,454
	(54.00)	(51.16)	(53.97)	(51.02)	(53.89)	(50.92)	(54.09)	(50.79)	(53.90)	(50.95)
Female	3,998	49,592	4,438	55,914	3,316	40,598	3,686	46,546	3,867	48,576
	(46.00)	(48.84)	(46.03)	(48.98)	(46.11)	(49.08)	(45.91)	(49.21)	(46.10)	(49.05)
Total	8,692	101,549	9,641	114,162	7,192	83,457	8,029	94,583	8,389	99,030
	(100)	(100)	(100)	(100)	(100)	(100)	(100)	(100)	(100)	(100)
Maternal Education										
No education	2,282	21,827	2,334	23,412	1,684	16,639	2,026	20,343	1,970	19,644
	(26.25)	(21.49)	(24.21)	(20.51)	(23.41)	(19.94)	(25.23)	(21.51)	(23.48)	(19.84)
Primary	1,168	13,192	1,300	14,943	939	10,786	1,108	12,528	1,113	12,941
	(13.44)	(12.99)	(13.48)	(13.09)	(13.06)	(12.92)	(13.80)	(13.25)	(13.27)	(13.07)
Secondary	4,351	53,741	4,997	61,558	3,796	45,325	4,111	50,627	4,410	53,878
	(50.06)	(52.92)	(51.83)	(53.92)	(52.78)	(54.31)	(51.20)	(53.53)	(52.57)	(54.41)
Higher	891	12,789	1,010	14,249	773	10,707	784	11,085	896	12,567
	(10.25)	(12.59)	(10.48)	(12.48)	(10.75)	(12.83)	(9.76)	(11.72)	(10.68)	(12.69)
Total	8,692	101,549	9,641	114,162	7,192	83,457	8,029	94,583	8,389	99,030
	(100)	(100)	(100)	(100)	(100)	(100)	(100)	(100)	(100)	(100)
Caste										
SC	1,917	22,510	2,100	24,803	1,563	18,066	1,711	20,625	1,800	21,385
	(23.05)	(23.07)	(22.99)	(22.81)	(22.77)	(22.63)	(22.58)	(22.96)	(22.61)	(22.61)
ST	1,774	18,448	2,119	23,586	1,545	16,295	1,757	19,194	1,829	19,587
	(21.33)	(18.91)	(23.19)	(21.69)	(22.51)	(20.41)	(23.19)	(21.36)	(22.97)	(20.99)
OBC	3,573	42,295	3,715	44,088	2,872	33,898	3,115	36,855	3,290	39,288
	(42.96)	(43.35)	(40.66)	(40.55)	(41.85)	(42.45)	(41.12)	(41.02)	(41.33)	(41.54)
Others	1,054	14,316	1,202	16,250	883	11,586	993	13,175	1,042	14,054
	(12.67)	(14.67)	(13.16)	(14.95)	(12.87)	(14.51)	(13.11)	(14.66)	(13.09)	(14.86)
Total	8,318	97,569	9,136	108,727	6,863	79,485	7,576	89,849	7,961	94,584
	(100)	(100)	(100)	(100)	(100)	(100)	(100)	(100)	(100)	(100)
Wealth Quintile										
Poorest	2,777	27,559	2,981	30,500	2,179	21,524	2,589	26,379	2,533	25,720
	(31.95)	(27.14)	(30.92)	(26.72)	(30.30)	(25.79)	(32.25)	(27.89)	(30.19)	(25.97)
Poorer	2,117	24,347	2,379	27,591	1,703	19,920	1,979	23,325	2,036	23,703
	(24.36)	(23.98)	(24.68)	(24.17)	(23.68)	(23.87)	(24.65)	(24.66)	(24.27)	(23.94)
Middle	1,702	20,860	1,928	23,758	1,488	17,757	1,583	19,271	1,722	20,982
	(19.58)	(20.54)	(20.00)	(20.81)	(20.69)	(21.28)	(19.72)	(20.37)	(20.53)	(21.19)
Richer	1,312	17,277	1,499	19,711	1,153	14,932	1,182	15,709	1,325	17,472
	(15.09)	(17.01)	(15.55)	(17.27)	(16.03)	(17.89)	(14.72)	(16.61)	(15.79)	(17.64)
Richest	784	11,506	854	12,602	669	9,324	696	9,899	773	11,153
	(9.02)	(11.33)	(8.86)	(11.04)	(9.30)	(11.17)	(8.67)	(10.47)	(9.21)	(11.26)
Total	8,692	101,549	9,641	114,162	7,192	83,457	8,029	94,583	8,389	99,030
	(100)	(100)	(100)	(100)	(100)	(100)	(100)	(100)	(100)	(100)
Place of residence										
Urban	1,419	17,010	1,563	19,061	1,223	14,368	1,281	15,692	1,399	16,868
	(16.33)	(16.75)	(16.21)	(16.70)	(17.01)	(17.22)	(15.95)	(16.59)	(16.68)	(17.03)
Rural	7,273	84,539	8,078	95,101	5,969	69,089	6,748	78,891	6,990	82,162
	(83.67)	(83.25)	(83.79)	(83.30)	(82.99)	(82.78)	(84.05)	(83.41)	(83.32)	(82.97)
Total	8,692	101,549	9,641	114,162	7,192	83,457	8,029	94,583	8,389	99,030
	(100)	(100)	(100)	(100)	(100)	(100)	(100)	(100)	(100)	(100)

**Fig 4:**
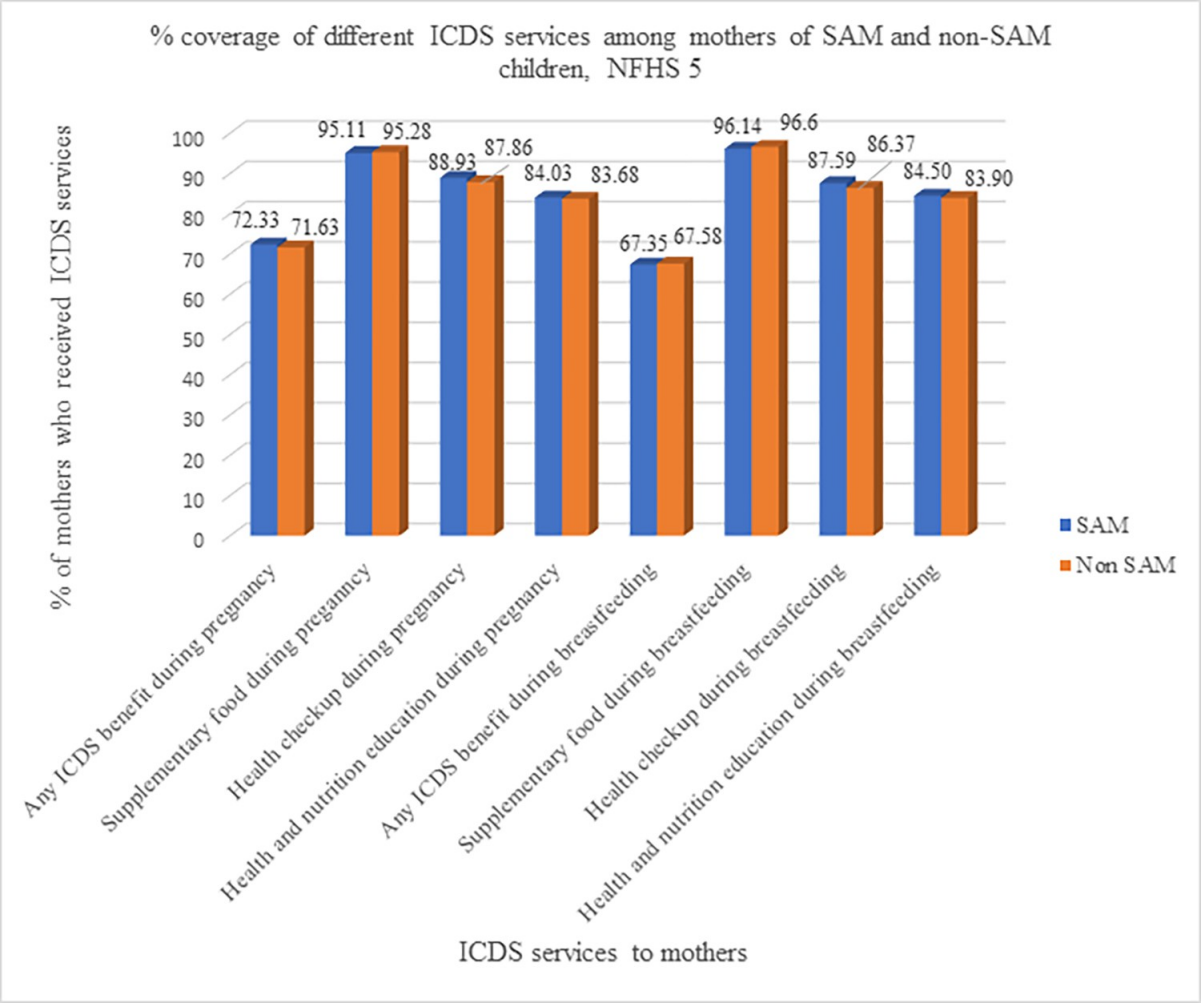
Percentage coverage of different ICDS services among mothers of SAM and Non-SAM children, NFHS 5.

Fig 4 shows the percentage coverage of different ICDS services among mothers of SAM and non-SAM children. It can be seen that the coverage of the various ICDS services among mothers of SAM and non-SAM children were almost identical Table 4 gives the coverage of different ICDS services for mothers of SAM and non-SAM children during their pregnancy and during their breastfeeding period. The different ICDS services include supplementary food, health check-up and health & nutrition education for the mothers. The coverage is higher among mothers of SAM children aged less than 3 years, male children, mothers with no or primary education, belonging to Scheduled Caste or Tribe and belonging to the poorest and poorer wealth quintile. The rest of the categories had better coverage of the ICDS services for mothers of non-SAM children than mothers of SAM children.

**Table 4:** Coverage of different ICDS services among mothers of SAM and Non-SAM children by background characteristics.

	During Pregnancy						During Breastfeeding					
Type of ICDS service →	Supplementary food		Health check-up		Health and nutrition education		Supplementary food		Health check-up		Health and nutrition education	
Type of child →	SAM (n,%)	Non-SAM (n,%)	SAM (n,%)	Non-SAM (n,%)	SAM (n,%)	Non-SAM (n,%)	SAM (n,%)	Non-SAM (n,%)	SAM (n,%)	Non-SAM (n,%)	SAM (n,%)	Non-SAM (n,%)
Background Characteristics ↓												
Age of child												
< 3 years	7,209 (68.35)	74,581 (58.64)	6,744 (68.39)	68,856 (58.70)	6,331 (67.94)	65,458 (58.60)	6,739 (67.93)	70,936 (58.34)	6,138 (67.91)	63,336 (58.27)	5,903 (67.69)	61,440 (58.19)
3–5 years	3,338 (31.65)	52,607 (41.36)	3,117 (31.61)	48,438 (41.30)	2,987 (32.06)	46,244 (41.40)	3,182 (32.07)	50,648 (41.66)	2,900 (32.09)	45,364 (41.73)	2,817 (32.31)	44,153 (41.81)
Total	10,547 (100)	127,188 (100)	9,861 (100)	117,294 (100)	9,318 (100)	111,702 (100)	9,921 (100)	121,584 (100)	9,038 (100)	108,700 (100)	8,720 (100)	105,593 (100)
Sex of child												
Male	5,725 (54.28)	65,141 (51.22)	5,341 (54.16)	61,013 (51.16)	5,052 (54.22)	57,090 (51.11)	5,361 (54.04)	62,157 (51.12)	4,860 (53.77)	55,594 (51.14)	4,691 (53.80)	53,941 (51.08)
Female	4,822 (45.72)	62,047 (48.78)	4,520 (45.84)	57,281 (48.84)	4,266 (45.78)	54,612 (48.89)	4,560 (45.96)	59,427 (48.88)	4,178 (46.23)	53,106 (48.86)	4,029 (46.20)	51,652 (48.92)
Total	10,547 (100)	127,188 (100)	9,861 (100)	117,294 (100)	9,318 (100)	111,702 (100)	9,921 (100)	121,584 (100)	9,038 (100)	108,700 (100)	8,720 (100)	105,593 (100)
Maternal Education												
No education	2,585 (24.51)	26,289 (20.67)	2,447 (24.81)	24,623 (20.99)	2,279 (24.46)	22,831 (20.44)	2,412 (24.31)	25,001 (20.56)	2,216 (24.52)	22,505 (20.70)	2,100 (24.08)	21,466 (20.33)
Primary	1,444 (13.69)	16,783 (13.20)	1,319 (13.38)	15,373 (13.11)	1,226 (13.16)	14,496 (12.98)	1,347 (13.58)	15,996 (13.16)	1,201 (13.29)	14,068 (12.94)	1,149 (13.18)	13,606 (12.89)
Secondary	5,439 (51.57)	68,516 (53.87)	5,071 (51.42)	62,690 (53.45)	4,844 (51.99)	60,308 (53.99)	5,164 (52.05)	65,688 (54.03)	4,701 (52.01)	58,518 (53.83)	4,586 (52.59)	57,256 (54.22)
Higher	1,079 (10.23)	15,600 (12.27)	1,024 (10.38)	14,608 (12.45)	969 (10.40)	14,067 (12.59)	998 (10.06)	14,899 (12.25)	920 (10.18)	13,609 (12.52)	885 (10.15)	13,265 (12.56)
Total	10,547 (100)	127,188 (100)	9,861 (100)	117,294 (100)	9,318 (100)	111,702 (100)	9,921 (100)	121,584 (100)	9,038 (100)	108,700 (100)	8,720 (100)	105,593 (100)
Caste												
SC	2,315 (23.19)	27,748 (22.88)	2,188 (23.27)	26,249 (23.38)	2,045 (23.06)	24,609 (23.03)	2,173 (23.12)	26,349 (22.72)	1,982 (23.00)	23,997 (23.07)	1,881 (22.63)	23,089 (22.86)
ST	2,284 (22.88)	25,911 (21.37)	2,030 (21.59)	21,710 (19.34)	1,997 (22.52)	21,636 (20.25)	2,174 (23.13)	24,901 (21.47)	1,909 (22.16)	20,584 (19.79)	1,887 (22.70)	20,655 (20.45)
OBC	4,129 (41.36)	49,736 (41.01)	4,016 (42.72)	47,851 (42.63)	3,731 (42.07)	45,003 (42.12)	3,882 (41.30)	47,837 (41.24)	3,676 (42.66)	44,255 (42.55)	3,515 (42.29)	42,642 (42.21)
Others	1,255 (12.57)	17,874 (14.74)	1,167 (12.41)	16,439 (14.65)	1,096 (12.36)	15,599 (14.60)	1,171 (12.46)	16,908 (14.58)	1,049 (12.18)	15,182 (14.60)	1,029 (12.38)	14,630 (14.48)
Total	9,983 (100)	121,269 (100)	9,401 (100)	112,249 (100)	8,869 (100)	106,847 (100)	9,400 (100)	115,995 (100)	8,616 (100)	104,018 (100)	8,312 (100)	101,016 (100)
Wealth Quintile												
Poorest	3,327 (31.54)	34,586 (27.19)	3,073 (31.16)	31,216 (26.61)	2,889 (31.00)	29,241 (26.18)	3,147 (31.72)	33,114 (27.24)	2,815 (31.15)	28,807 (26.50)	2,695 (30.91)	27,604 (26.14)
Poorer	2,577 (24.43)	30,981 (24.29)	2,388 (24.22)	28,128 (23.98)	2,255 (24.20)	26,649 (23.86)	2,422 (24.41)	29,497 (24.26)	2,186 (24.19)	26,008 (23.93)	2,108 (24.17)	25,217 (23.88)
Middle	2,119 (20.09)	26,265 (20.65)	1,987 (20.15)	24,433 (20.83)	1,856 (19.92)	23,530 (21.06)	1,999 (20.15)	25,176 (20.71)	1,827 (20.21)	22,753 (20.93)	1,754 (20.11)	22,300 (21.120
Richer	1,608 (15.25)	21,636 (17.01)	1,529 (15.51)	20,213 (17.23)	1,483 (15.92)	19,540 (17.49)	1,514 (15.26)	20,702 (17.03)	1,409 (15.59)	18,821 (17.31)	1,387 (15.91)	18,512 (17.53)
Richest	916 (8.68)	13,810 (10.86)	884 (8.96)	13,304 (11.34)	835 (8.96)	12,742 (11.41)	839 (8.46)	13,095 (10.77)	801 (8.86)	12,311 (11.33)	776 (8.90)	11,960 (11.33)
Total	10,547 (100)	127,188 (100)	9,861 (100)	117,294 (100)	9,318 (100)	111,702 (100)	9,921 (100)	121,584 (100)	9,038 (100)	108,700 (100)	8,720 (100)	105,593 (100)
Place of residence												
Urban	1,667 (15.81)	20,612 (16.21)	1,600 (16.23)	19,394 (16.53)	1,550 (16.63)	18,754 (16.79)	1,575 (15.88)	19,573 (16.25)	1,474 (16.31)	18,124 (16.67)	1,444 (16.56)	17,811 (16.87)
Rural	8,880 (84.19)	106,576 (83.79)	8,261 (83.77)	97,900 (83.47)	7,768 (83.37)	92,948 (83.21)	8,346 (84.12)	101,831 (83.75)	7,564 (83.69)	90,576 (83.33)	7,276 (83.44)	87,782 (83.13)
Total	10,547 (100)	127,188 (100)	9,861 (100)	117,294 (100)	9,318 (100)	111,702 (100)	9,921 (100)	121,584 (100)	9,038 (100)	108,700 (100)	8,720 (100)	105,593 (100)

The Odds Ratio based on the logistic regression model is presented in Table 5. Results indicate that children’s age had a significant relationship with the outcome variable. Children in the age group 1–2 years (Odds Ratio [OR] 1.76; CI: (1.51, 2.04)) and 2–3 years (OR 1.34; CI: (1.15, 1.55)) had a greater likelihood of receiving ICDS services. Female SAM children were less likely to be covered under ICDS. Birth order and religion of the child had no significant association with SAM coverage under ICDS. ST, OBC and children of other caste had lower chances of receiving ICDS services than the scheduled caste (SC) children. Primary and secondary education of mothers had higher odds ratio ([OR] 1.07 and 1.003 respectively) than mothers with no education. The chances of SAM children being covered under ICDS services are greater for those in the poorer and middle wealth quintiles, and having institutional deliveries as compared to their counterparts, though these associations were not statistically significant. SAM children living in the rural areas had a significantly higher odds of being covered under ICDS service (OR 1.57; CI: (1.35, 1.82)) than those living in the urban areas. SAM children whose mothers received ICDS benefits during pregnancy and breastfeeding had a significantly higher chance of receiving ICDS services ([OR] 5.27 and 5.95 respectively). Also, children of those households exposed to mass media were 1.15 times more likely to be covered under ICDS than those who were not exposed to mass media.

**Table 5:** Determinants of SAM coverage under ICDS and total coverage under ICDS, NFHS 5.

Variables ↓	Determinants for ICDS Coverage		Determinants for SAM coverage under ICDS	
	OR (CI)	p-value	OR (CI)	p-value
Age of child				
0–1 year (Ref)	1.00		1.00	
1–2 years	1.65 (1.58–1.72)	p<0.01	1.76 (1.51–2.04)	p<0.01
2–3 years	1.23 (1.17–1.28)	p<0.01	1.34 (1.15–1.55)	p<0.01
3–4 years	0.77 (0.74–0.80)	p<0.01	0.75 (0.66–0.88)	p<0.01
4–5 years	0.50 (0.48–0.52)	p<0.01	0.57 (0.48–0.66)	p<0.01
Sex of child				
Male (Ref)	1.00		1.00	
Female	1.02 (0.99–1.05)	0.115	0.99 (0.90–1.09)	0.823
Birth Order				
Birth Order 1–2 (Ref)	1.00		1.00	
Birth Order 3–4	1.01 (0.98–1.05)	0.454	0.96 (0.84–1.09)	0.532
Birth Order 4+	1.00 (0.94–1.06)	0.981	0.89 (0.72–1.11)	0.31
Religion				
Hindu (Ref)	1.00		1.00	
Muslim	0.96 (0.91–1.01)	0.131	1.04 (0.89–1.22)	0.633
Christian	1.09 (0.98–1.20)	0.101	0.94 (0.71–1.25)	0.658
Others	0.96 (0.88–1.05)	0.38	0.74 (0.54–1.01)	0.06
Caste				
Scheduled Caste (Ref)	1.00		1.00	
Scheduled Tribe	1.01 (0.95–1.08)	0.691	0.97 (0.80–1.18)	0.791
Other Backward Class	0.95 (0.92–0.99)	0.022	0.84 (0.73–0.97)	0.019
Others	0.92 (0.88–0.97)	0.001	0.81 (0.68–0.97)	0.02
Birth Interval				
< 24 months (Ref)	1.00		1.00	
24–35 months	0.98 (0.94–1.03)	0.481	1.06 (0.90–1.26)	0.479
36–47 months	1.01 (0.96–1.06)	0.72	1.10 (0.91–1.33)	0.32
48–59 months	0.98 (0.92–1.04)	0.537	1.06 (0.84–1.33)	0.638
60+ months	0.91 (0.88–0.96)	p<0.01	0.97 (0.83–1.13)	0.692
Mother’s education				
No education (Ref)	1.00		1.00	
Primary	1.06 (1.01–1.11)	0.025	1.07 (0.90–1.28)	0.429
Secondary	1.03 (0.99–1.070	0.116	1.00 (0.87–1.16)	0.97
Higher	0.92 (0.86–0.97)	0.003	0.92 (0.74–1.13)	0.422
Wealth Index				
Poorest (Ref)	1.00		1.00	
Poorer	0.99 (0.96–1.04)	0.909	1.04 (0.90–1.20)	0.619
Middle	0.96 (0.92–1.01)	0.147	1.10 (0.92–1.31)	0.284
Richer	0.91 (0.86–0.97)	0.001	1.02 (0.83–1.24)	0.87
Richest	0.79 (0.74–0.84)	p<0.01	0.94 (0.74–1.20)	0.617
Place of residence				
Urban (Ref)	1.00		1.00	
Rural	1.46 (1.39–1.54)	p<0.01	1.57 (1.35–1.82)	p<0.01
Place of delivery				
Non-institutional (Ref)	1.00		1.00	
Public institutional	1.14 (1.09–1.18)	p<0.01	1.13 (0.97–1.32)	0.116
Private Institutional	1.03 (0.96–1.09)	0.292	1.02 (0.85–1.25)	0.789
Other	1.18 (0.89–1.58)	0.274	2.62 (0.72–9.50)	0.144
Mother received ICDS benefit during pregnancy				
No (Ref)	1.00		1.00	
Yes	4.51 (4.34–4.68)	p<0.01	5.27 (4.57–6.09)	p<0.01
Mother received ICDS benefit during breastfeeding				
No (Ref)	1.00		1.00	
Yes	4.91 (4.73–5.09)	p<0.01	5.95 (5.16–6.85)	p<0.01
Media Exposure				
No (Ref)	1.00		1.00	
Yes	1.16 (1.12–1.21)	p<0.01	1.15 (1.02–1.31)	0.027

Table 5 also gives a comparison of the likelihood of SAM coverage under ICDS and ICDS coverage for all children. As can be seen, the significant determinants of SAM coverage under ICDS were almost similar with the determinants of ICDS coverage for all children. For example, all children in the age group 1–2 years and 2–3 years had better likelihood of getting ICDS services (OR 1.65 and 1.23 respectively) than their counterparts as were SAM children of the same age group (OR 1.76 and 1.34). Similarly, children residing in rural areas had higher chances of receiving ICDS benefits (OR 1.46) as were children suffering from SAM living in rural areas (OR 1.57). Mothers receiving ICDS benefits during pregnancy and breastfeeding were also significant factors in SAM coverage under ICDS and ICDS coverage for all children.

A model applied without any covariate (the null model) showed a significant amount of variation in SAM coverage under ICDS across the PSU, district, and state levels (Table 6). Based on the variance partitioning coefficient (VPC) values, 27%, 16% and 57% of the total variance in SAM coverage under ICDS were attributable to differences between the state, district and PSU levels, respectively. After including all the covariates in the null model, the VPC values showed that 14.6%, 12.4% and 73% of the total variance in SAM coverage under ICDS were attributable to variations between the state, district and PSU levels respectively. Thus, state and district level focus on ICDS program implementation is necessary to reduce such disparities at these higher administrative units.

**Table 6:** Variance estimates and VPC across PSU, district, and state level.

Random effect parameters	Null Model		Adjusted Model	
	Variance (SE)	95% CI	Variance (SE)	95% CI
State-level variance	0.709 (0.191)	0.417, 1.203	0.124 (0.046)	0.059, 0.257
District level variance	0.419 (0.054)	0.325, 0.541	0.106 (0.031)	0.059, 0.189
PSU level variance	1.489 (0.155)	1.214, 1.828	0.619 (0.124)	0.418, 0.918
State-level VPC (%)	0.271 (27.1%)		0.146 (14.6%)	
District level VPC (%)	0.16 (16%)		0.124 (12.4%)	
PSU level VPC (%)	0.569 (56.9%)		0.73 (73%)	

Note: The second model was adjusted for all the socio-economic and demographic correlates.

## Discussion

The salient findings from this study are as follows: First, there is no evidence that ICDS is more efficient in identifying and covering SAM children than non-SAM children. It should improve targeting of SAM children under the program in high burden geographies. Second, the burden of SAM is more among older children (3+ age group). This group is generally neglected and participation in the ICDS programme comes down drastically for this age group. Third, despite special provisioning in place for SAM children, coverage of different ICDS services was similar to that of non-SAM children, and were in fact lower than non-SAM children for some categories. The significant determinants of higher SAM coverage under ICDS were age and caste of the child, place of residence and mothers’ utilization of ICDS benefits during pregnancy and breastfeeding. This suggests that improving coverage of ICDS services among pregnant and lactating mothers would increase the coverage of ICDS services among SAM children.

Identifying children suffering from SAM and providing treatment is a major concern in the nutritional programmes. The Integrated Child Development Services-Common Application Software (ICDS-CAS) was launched to digitise data and develop a real-time monitoring system for those who are benefiting under the National Nutrition Mission (POSHAN Abhiyaan). However, until 2019, only around 60% of the Anganwadi Centres (AWCs) across the country were equipped with the ICDS-CAS [33]. Studies conducted across the states have revealed high levels of under-reporting of SAM, mainly due to lack of capacity building among the *Anganwadi* workers (AWWs), their lack of training and unavailability of growth monitoring devices. Fear of reputational harm to the district leads to pressure from administrative higher authorities on the AWWs to under-report the prevalence of SAM [34–36]. Precise anthropometry is required to correctly identify, diagnose and manage children with SAM.

Capacity building of the AWWs is a crucial component in identifying SAM children, with specific focus needed on the appropriate use of growth monitoring devices. Apart from the individual capacity of the AWWs, there are other multiple levels of factors that hamper the effectiveness of the ICDS services in targeting SAM. Some of these include inadequate infrastructure and financing, governance failure, household migration for economic reasons, delayed salaries and high workload of the AWWs [37–39]. Under NHM, the Nutritional Rehabilitation Centres (NRCs) were set up where children with Severe Acute Malnutrition (SAM) are admitted and managed. A steady linkage with ICDS identifies the severely malnourished children in the community and refers them to the NRCs. However, recent data suggests that only about 20% of the SAM children could be provided treatment under the NRCs, mainly due to poor functioning of the NRCs and high drop-out rates [40, 41].

ICDS service utilization among SAM children has been found to be lower among children in the age group 3–5 years. This is consistent with other studies that have shown that the majority of the beneficiaries of the ICDS programme are children in the age group 0–3 years [42]. The diet provided to 3+ year old children in ICDS centres is not of high quality or composition value in some states. For instance, a study in rural Bihar shows that the energy content in the food provided to children above 3 years is enough to meet only 10–14 percent of the daily requirements. This is also true for protein and iron intakes, with the food provided at ICDS centre meeting only 35–40 percent, and 16–21 percent of the daily requirements respectively [43]. Studies have also shown that this drop in ICDS coverage is more among the poor and the SAM burden groups. It has been seen that the states with the worst malnutrition have the lowest levels of ICDS programme coverage and that the relatively better-off households are more likely to use ICDS services than poorer communities [44, 45]. Health programmes do not focus much on children in the age group 3–6 years. For instance, the Home-based care for young children (HBYC) programme is devised for children 3–15 months old and home-based newborn care (HBNC) programme for newborns, whereas 3+ children are relatively ignored. Under the Home-based care for young children (HBYC), a series of structured home visits are made by ASHA workers supported by AWWs to homes of children 3–15 months old in order to counsel them on key domains of nutrition, health, early child development (ECD) and WASH practices [46]. Under the Home-based newborn care (HBNC), the ASHA workers provide home based care to children born until 42 days after birth of the child [46]. Health programmes focusing on children above 3 years is the need of the hour. This is more important because of rapid deterioration in nutritional status of children in 3–5 year age group.

SAM is a marker of high mortality. Studies across Asia, Africa and South America have showed that children with severe wasting have a nine-fold increased mortality rates as compared to children with WHZ > -1 [47]. Before the advent of community-based management of acute malnutrition (CMAM), WHO estimated that almost 10% to 20% of children with SAM generally die within 2–3 months without treatment [48–50]. But these studies were conducted about 15 years ago, and mostly in African countries, and did not take into account whether the child had medical complications or not. The survival of SAM children in India is, however, better and the share of under-five deaths accountable to SAM has gradually declined. A study conducted in rural Jharkhand and Odisha revealed that SAM carried a lower-case fatality rate (1.2%) than WHO estimates (10%-20%) [51]. This clearly shows the relevance of targeting SAM for child health and survival.

SAM participation can be improved with improved child diets at the Anganwadi centres. Often the food is considered of poor quality in terms of both nutrition and taste. Sometimes, the food deteriorates and is found unfit for consumption, and many digestive problems are attributed to it [52]. Several reports in the past have highlighted the fact that there is a large gap in the feeding program of the ICDS and that food supplies are also erratic [53]. As per the ICDS WCD guidelines, the SAM children are given special meals. As per the revised norms, a SAM child gets a diet that consists of 800 kcal of energy and 20–25 grams of protein daily [54]. The SAM children also have a higher cost norm of Rs. 9.00 per beneficiary per day as compared to Rs. 6.00 for other beneficiaries. This programme also allocates Rs 12.00 per beneficiary per day for the SAM children aged 6 months-72 months as per reforms in 2018. The ICDS had a budget of Rs 20,532 crores and Rs 20,105 for the financial year 2020–21 and 2021–22, respectively [55]. The funding commitments of the ICDS is shared between the Central and State governments. Currently, the sharing pattern for supplementary nutrition between the Center and States is 90:10 for North-Eastern states and 50:50 for other States/UTs. Improving the concurrent monitoring and supervision activities on dietary support for the SAM children is also an important area for policy action. For example, a study by Bredenkamp and Akin, 2004 gave evidences of empirical association between those states in which AWCs have been identified as better performers and those that receive community support for ICDS in the form of financial contributions from the panchayat. It has been found that only 25% of states receive support from the panchayat leaders, and this support is mainly in the form of providing space for AWC or recruiting beneficiaries. With the kind of decentralisation that is underway in India, there is ample scope for involving village communities much more actively in the implementation of the ICDS programme [42].

Improvement in the coverage of SAM children under the ICDS programme over the last couple of years is evident but there is a further need to boost the quality and components of services. The POSHAN Abhiyaan and related momentum on nutrition may have led to improvement in ICDS use but implementation with an equity focus is necessary for sustained action. Interventions at hard-to-reach geographies and vulnerable communities continue to be much relevant priorities. Besides, policymaking should focus on improved monitoring through administrative data at the lower levels that includes ICDS centres, villages, and blocks. This can help to strengthen programme research and support efficient targeting and monitoring mechanisms for SAM children.

Further research can be done to explore factors that predict provision and receipt of ICDS services among households and frontline workers. The education and experience of the Anganwadi workers, incentives provided to them, their knowledge on nutrition related topics may have an impact on the quality of ICDS services provided to children. Secondary data in India generally do not provide any information on these issues. So, primary data is required to asses these important aspects of ICDS service delivery and uptake.

## Conclusion

This study examines the extent of ICDS service utilization among (SAM) children in India along with the factors that determine SAM coverage under ICDS. Results show that SAM do not receive full coverage under ICDS and do not get special attention as they should, given their high mortality risks. There is ample scope for the ICDS to improvise ways to efficiently reach out to SAM children through convergence mechanisms and better identification and nutrition support programmes. Along with good food supplementation that is identified as an essential service under ICDS, it is also critical that Anganwadi workers (AWWs) are provided with adequate training for their skill development. Results from econometric analysis suggests that children in the lower age group had better chances of ICDS service utilization. It is necessary to include children in the 3+ years age group. Given the all-around focus of ICDS on child health and development, it is critical to strengthen the program and demonstrate impact of coverage through lower SAM burden as well as reduced child mortality. Strengthening research and supervision based on concurrent monitoring and administrative data along with better community participation and improvements in coverage and service provisioning can provide insights to improve SAM coverage under ICDS and reduce the burden of child mortality in India.

## Limitations of the study

This study was, however, not free from certain limitations. First, NFHS provides cross-sectional data, which does not allow causal interpretation between the outcome and observed correlates. Secondly, some children who may have been SAM may have accessed ICDS, but as per current measurement they may not be SAM. Hence, there is a possibility of a selection bias. Besides, the ICDS use definition categories are much broader. Not all families regularly use the ICDS services, resulting in a lower coverage of the programme. This may influence the magnitude of the problem and the observed associations.
